# Cardiovascular Risk Factors in Subclinical Hypothyroidism: A Case Control Study in Nepalese Population

**DOI:** 10.1155/2015/305241

**Published:** 2015-10-07

**Authors:** Rajendra KC, Saroj Khatiwada, Kishun Deo Mehta, Pratikshya Pandey, Madhab Lamsal, Shankhar Majhi

**Affiliations:** ^1^Department of Medical Laboratory Technology, Modern Technical College, Satdobato, Lalitpur, Nepal; ^2^Department of Pharmacy, Central Institute of Science and Technology (CIST) College, Pokhara University, Kathmandu, Nepal; ^3^Department of Biochemistry, B.P. Koirala Institute of Health Sciences, Dharan, Nepal; ^4^Department of Microbiology, Tribhuvan University, Kirtipur, Nepal

## Abstract

*Objectives*. To assess cardiovascular risk factors in Nepalese population with subclinical hypothyroidism as compared to age and sex matched controls. *Materials and Methods*. A case control study was conducted among 200 subjects (100 subclinical hypothyroid and 100 euthyroid) at B.P. Koirala Institute of Health Sciences, Dharan, Nepal. Demographic and anthropometric variables including systolic and diastolic blood pressure (BP) were taken. Blood samples were assayed for serum free triiodothyronine (fT3), free thyroxine (fT4), thyroid stimulating hormone (TSH), total cholesterol (TC), high density lipoprotein cholesterol (HDL-C), low density lipoprotein cholesterol (LDL-C), and high sensitivity C reactive protein (hs-CRP). *Results*. Subclinical hypothyroid patients had significantly higher diastolic BP, total cholesterol, LDL cholesterol, and hs-CRP than controls. The odds ratio of having hypercholesterolemia (>200 mg/dL), low HDL cholesterol (<40 mg/dL), undesirable LDL-cholesterol (>100 mg/dL), high hs-CRP (>1 mg/L), and high diastolic BP (>80 mmHg) and being overweight (BMI ≥ 23 Kg/m^2^) in subclinical hypothyroidism was 2.29 (95% CI; 1.2–4.38, *p* = 0.011), 1.73 (95% CI; 0.82–3.62, *p* = 0.141), 3.04 (95% CI; 1.66–5.56, *p* < 0.001), 2.02 (95% CI; 1.12–3.64, *p* = 0.018), 3.35 (95% CI; 1.72–6.55, *p* < 0.001), and 0.9 (95% CI; 0.48–1.67, *p* = 0.753), respectively, as compared to controls. *Conclusion*. Subclinical hypothyroid patients are associated with higher risk for cardiovascular disease than euthyroid subjects.

## 1. Introduction

Thyroid disorders are the commonest endocrine disorders worldwide [1]. Thyroid dysfunction is a graded phenomenon, which progresses from early (mild or subclinical thyroid dysfunction) to more advanced forms (overt thyroid dysfunction). Subclinical hypothyroidism is characterized by high thyroid stimulating hormone (TSH) and normal triiodothyronine (T3) and thyroxine (T4) with no significant clinical symptoms [2]. Subclinical hypothyroidism has been diagnosed more frequently since the widespread use of TSH screening with high-sensitivity assays. However, the treatment and management of these patients are still a matter of debate [2, 3].

Thyroid hormones regulate a wide array of metabolic parameters including lipoprotein metabolism and cardiovascular disease (CVD) risk factors, thus influencing overall CDV risk [4]. The major cardiovascular risk factors include central obesity, glucose intolerance or type 2 diabetes, dyslipidemia, high low density lipoprotein cholesterol (LDL-C) levels, and hypertension [5]. There are data both against and in favor of the role of subclinical thyroid dysfunction as a risk factor for cardiovascular disease, and several reports have demonstrated a positive correlation between TSH levels and several cardiovascular risk factors, even in healthy euthyroid populations. Thus, the effect of increased TSH on CVD risk factors is still unresolved [6].

Subclinical hypothyroidism (SH) affects 3–15% of the adult population [7]. If subclinical hypothyroidism significantly increases the risk for CVD then a great proportion of SH patients could be prevented from developing CVD by treating for the subclinical hypothyroid condition. In a hospital based study in Nepal, the prevalence of subclinical hypothyroidism was 20.42%, which is much higher compared to the other parts of the world [8]. A small study in Nepalese population revealed that cases of subclinical hypothyroidism are at risk of dyslipidemia as compared to controls [9]. Scarce data is available about CVD risk factors in Nepalese population with subclinical hypothyroidism. Hence, the present study was undertaken to investigate the cardiovascular risk factors in Nepalese subjects with subclinical hypothyroidism as compared to matched euthyroid controls.

Subjects with subclinical hypothyroidism as cases and euthyroid subjects as controls were selected and comparative study of CVD risk factors among the groups was done.

## 2. Materials and Methods

A matched case-control study was conducted among 200 subjects (100 subclinical hypothyroid patients as cases and 100 euthyroid subjects as controls) from 2012 to 2013 at B.P. Koirala Institute of Health Sciences, Dharan, Nepal. Subclinical hypothyroid patients (100 cases) and 100 age and sex matched euthyroid subjects as controls were selected during study period. Patients with normal free T3 (4.0–8.3 pmol/L), free T4 (9.0–20.0 pmol/L), and TSH level above 5 mIU/L (0.25–5 mIU/L) were considered as having subclinical hypothyroidism. The patients diagnosed as hyperthyroid or hypothyroid, on medication that affects thyroid hormone levels, known cases of CVD, patients using oral contraceptives, hormonal preparations, and lipid lowering agents, were excluded. Similarly patients with diabetes mellitus, renal failure, hypertension, and any other chronic illness were also excluded. The consent was taken from each subject and the ethical approval for the study was provided by institute review board of B.P. Koirala Institute of Health Sciences, Dharan.

Age, sex, weight, height, resting systolic, and diastolic blood pressure of each subject were noted using a questionnaire. Overnight fasting venous blood samples (5 mL) were collected in plain vials, and the serum was separated and stored at −20°C until analysis. Serum free T3, free T4, and TSH were measured by using fluorescent immunoassay (VIDAS, Biomerieux SA, France). Similarly, total cholesterol was measured by enzymatic method (CHOD-PAP) using kit from AGAPPE diagnostics. Low density lipoprotein cholesterol (LDL-C) and high density lipoprotein cholesterol (HDL-C) were estimated by homogeneous, direct method using kit from Gesan by Biolyzer 100 (semiautoanalyzer), and high sensitive C reactive protein (hs-CRP) was measured by enzyme immunoassay using kit from Diagnostics Biochem Canada (DBC) and the reading was taken using LabLife ER 2007 plate reader. Ratio of total cholesterol to HDL cholesterol (TC/HDL-C) and ratio of LDL cholesterol to HDL cholesterol (LDL-C/HDL-C) were calculated.

The data generated from study were entered in MS excel and analyzed using SPSS software version 11.0. The data was expressed as mean ± SD values except for hs-CRP. Independent *t*-test, Mann-Whitney test, and chi square test were applied at 95% confidence interval to test for statistical significance. Pearson correlation and Spearman's Rho correlation analysis was done to study correlation among variables. A *p* value less than 0.05 at 95% confidence interval was considered statistically significant.

## 3. Results

Number of males (mean age 42.96 ± 12.33 years) and number of females (mean age 40.57 ± 10.46 years) were 24 and 76, respectively, for both case and control groups. The mean age was 41.1 ± 10.9 years for both cases and controls. Various parameters of the study population are shown in Table 1. Subclinical hypothyroid patients had significantly higher diastolic BP, total cholesterol, LDL cholesterol, TC/HDL-C, LDL-C/HDL-C, and hs-CRP than controls. Subclinical hypothyroid patients with TSH >10 mIU/L had significantly higher BMI, systolic BP, diastolic BP, free T3, free T4, total cholesterol, LDL-C, TC/HDL-C, LDL-C/HDL-C, and hs-CRP than subclinical hypothyroid patients with TSH <10 mIU/L as shown in Table 1.

In the case group, males had significantly higher diastolic BP (84.58 ± 7.08 mmHg versus 81.29 ± 5.47 mmHg, *p* = 0.045), LDL-C (117.33 ± 33.81 mg/dL versus 99.83 ± 23.94 mg/dL, *p* = 0.025), and hs-CRP (1.74 mg/L (0.63–4.02) versus 0.77 mg/L (0.34–1.6), *p* = 0.009) than females, whereas females had significantly higher HDL-C (48.33 ± 6.66 mg/dL versus 38.21 ± 5.74 mg/dL, *p* < 0.001) than males. However, no significant difference was observed among BMI (25.08 ± 2.55 Kg/m^2^ versus 24.33 ± 2.19 Kg/m^2^, *p* = 0.161), systolic BP (123.33 ± 8.03 mmHg versus 120.24 ± 5.69 mmHg, *p* = 0.089), and total cholesterol (211.67 ± 36.89 mg/dL versus 195.75 ± 28.95 mg/dL, *p* = 0.062) of males and females, respectively, in case group. Similarly, in control (euthyroid group), males had significantly higher BMI (25.17 ± 1.3 Kg/m^2^ versus 23.79 ± 1.66 Kg/m^2^, *p* < 0.001), systolic BP (123.83 ± 5.46 mmHg versus 120.71 ± 6.09 mmHg, *p* = 0.027), LDL-C (103.5 ± 24.99 mg/dL versus 91.76 ± 25.08 mg/dL, *p* = 0.048), and hs-CRP (1.23 mg/L (0.59–2.52) versus 0.44 mg/L (0.28–0.75), *p* = 0.001) than females, whereas females had significantly higher HDL-C (50.09 ± 5.19 mg/dL versus 39 ± 4.03 mg/dL, *p* < 0.001) than males. Diastolic BP (80.92 ± 4.56 mmHg versus 78.55 ± 6.38 mmHg, *p* = 0.096) and total cholesterol (193.25 ± 27.85 mg/dL versus 181.43 ± 31.31 mg/dL, *p* = 0.10) were similar in both males and females, respectively, in control group.

Higher number of subclinical hypothyroid cases had total cholesterol >200 mg/dL (35 versus 19, *p* = 0.016), diastolic BP >80 mmHg (39 versus 16, *p* < 0.001), LDL-C >100 mg/dL (49 versus 24, *p* < 0.001), hs-CRP >1 mg/L (44 versus 28, *p* = 0.027), and HDL-C <40 mg/dL (22 versus 14, *p* = 0.197) compared to euthyroid controls except for BMI ≥23 Kg/m^2^ (71 versus 73, *p* = 0.875), respectively. TSH had significant positive correlation with BMI, systolic BP, diastolic BP, total cholesterol, LDL cholesterol, TC/HDL-C, LDL-C/HDL-C, and hs-CRP except for HDL-C among the cases. However, similar results were not observed for controls as shown in Table 2.

The odds ratio of having hypercholesterolemia (>200 mg/dL), low HDL cholesterol (<40 mg/dL), undesirable LDL-cholesterol (>100 mg/dL), high hs-CRP (>1 mg/L), and high diastolic BP (>80 mmHg) and being overweight (BMI ≥23 Kg/m^2^ for Asians) in subclinical hypothyroidism was 2.29 (95% CI; 1.2–4.38, *p* = 0.011), 1.73 (95% CI; 0.82–3.62, *p* = 0.141), 3.04 (95% CI; 1.66–5.56, *p* < 0.001), 2.02 (95% CI; 1.12–3.64, *p* = 0.018), 3.35 (95% CI; 1.72–6.55, *p* < 0.001), and 0.9 (95% CI; 0.48–1.67, *p* = 0.753), respectively, as compared to controls.

## 4. Discussion

Cardiovascular diseases are the major cause of death worldwide and it has significant health related costs. A number of CVD risk factors can be modified thereby decreasing the CVD risk [10]. Subclinical hypothyroidism, a mild thyroid dysfunction, has been reported increasingly all over the globe. The Wickham Survey and the Colorado study have shown prevalence of SH in 7.5% males and 3.1% females in general population [11]. Subclinical hypothyroidism has clinical importance because of its high prevalence (4–20%), the risk of progression to overt hypothyroidism, and consequences associated with cardiac and lipid abnormalities [12]. A number of studies have reported that subclinical hypothyroidism is associated with an increased risk of coronary heart disease (CHD) and there appears to be a significant increase in a cluster of metabolic CVD risk factors among people with subclinical hypothyroidism [13, 14]. Similar to previous studies, we found preponderance of risk factors for CVD in SH as compared to control. We observed significantly higher level of diastolic BP, total cholesterol, LDL cholesterol, TC/HDL-C, LDL-C/HDL-C, and hs-CRP in SH as compared to control. High diastolic BP observed in our study was consistent with that of study conducted in Israel, where diastolic blood pressure was 82 versus 75 mmHg in SH versus control women [15]. Study by Sharma et al. also demonstrated that patients with subclinical hypothyroidism had significantly higher levels of serum hs-CRP, Lp (a), total cholesterol, and LDL-C when compared to same parameters of controls [16]. Similar to finding of Kvetny et al., we found that patients with SH have low grade of inflammation as indicated by raised hs-CRP level compared to normal subjects [17]. This suggests for an association between thyroid function and low grade inflammation. Our finding reveals that patients with subclinical hypothyroidism had substantially increased risk of developing hypercholesterolemia and diastolic hypertension and having low HDL, undesirable LDL-C, and high hs-CRP. The alterations of lipids parameters can lead to development of atherosclerosis, which has serious consequences like development of coronary artery diseases and stroke [9]. A study by Hueston and Pearson, however, reported that hypothyroidism was not related to elevations in cholesterol levels, LDL levels, and triglyceride levels or to a low HDL level [18].

In our study population, 30 subclinical hypothyroid patients had TSH >10 mIU/L. It is strongly recommended that all patients with SH and a serum TSH level above 10 mIU/L should be treated with levothyroxine [19]. We observed higher risk for CVD in SH patients with TSH >10 mIU/L than those with TSH <10 mIU/L. Rise in TSH level >10 mIU/L among subclinical hypothyroid patients was associated with further increase in cardiovascular risk factors. Similar to the findings of Sharma et al., we observed a significant positive correlation between TSH and hs-CRP, LDL-C, and TC in subjects with subclinical hypothyroidism [16]. Study by Terán and Calle also showed that TSH levels have statistically significant association with total cholesterol and LDL levels but are not a good clinical predictor in this process [20]. Study by Wang et al. however did not report any significant correlation of TSH with CVD risk factors in subclinical hypothyroid patients [3]. High diastolic BP, hypercholesterolemia, low HDL-C, undesirable LDL-C, and high hs-CRP were much common in SH subjects compared to control. Hueston and Pearson also found that higher percentage of subclinical hypothyroid patients had elevated total cholesterol and LDL cholesterol and decreased HDL compared to control [18].

We observed that subclinical hypothyroid males have higher CVD risk than subclinical hypothyroid females, as indicated by the significantly high diastolic BP, LDL-C, and hs-CRP in males compared to females. Females, however, possessed significantly high level of HDL-C, which further decreases CVD risks. However, study by Yadav et al. had observed significantly higher total cholesterol and LDL-C in subclinical hypothyroid females than males [9]. Our findings suggest that assessment of TSH in patient with dyslipidemia may provide additional benefit for the management of CVD patients. However, in a study in subclinical hypothyroid by Kim et al., it was found that the additional assessment of serum TSH levels provided little incremental benefit for the prediction of cardiovascular risk [21].

From our results, it can be speculated that diastolic hypertension, atherogenic lipid profile, and low grade inflammation might increase risk of developing CVD in subjects with SH. However, we did follow up the SH patients for future CVD development. Since subclinical hypothyroidism is common in Nepalese population, the management of SH in such population might reduce the cardiovascular diseases burden.

In conclusion, the present study finds higher preponderance of CVD risk factors in subclinical hypothyroidism patients as compared to euthyroid subjects, and subclinical hypothyroid patients have significantly higher risk of having hypercholesterolemia, undesirable LDL cholesterol, high hs-CRP, and high diastolic BP than those with normal thyroid function.

## Figures and Tables

**Table 1:** Characteristics of the study subjects.

Variables	Subclinical hypothyroid (SH) (cases) (*n* = 100)				Euthyroid (controls) (*n* = 100)	*p* value (total)
	SH with TSH <10 mIU/L (*n* = 70)	SH with TSH ≥10 mIU/L (*n* = 30)	*p* value	Total (*n* = 100)		
Age (years)	42.23 ± 11.15	38.60 ± 10.07	0.202	41.14 ± 10.92	41.14 ± 10.92	1.0
BMI (Kg/m^2^)	23.87 ± 1.91	26.01 ± 2.51	<0.001	24.51 ± 2.32	24.11 ± 1.66	0.422
Systolic BP (mmHg)	119.6 ± 5.98	124.2 ± 6.37	<0.001	120.98 ± 6.42	121.46 ± 6.06	0.457
Diastolic BP (mmHg)	80.66 ± 5.86	85.4 ± 5.09	<0.001	82.08 ± 6.03	79.12 ± 6.05	<0.001
Free T3 (pmol/L)	4.82 ± 0.48	4.32 ± 0.3	<0.001	4.67 ± 0.49	5.11 ± 0.58	<0.001
Free T4 (pmol/L)	10.54 ± 0.85	9.47 ± 0.28	<0.001	10.22 ± 0.88	12.06 ± 2.0	<0.001
TSH (mIU/L)	7.73 ± 1.14	14.09 ± 3.48	<0.001	9.63 ± 3.61	2.35 ± 1.07	<0.001
Total cholesterol (mg/dL)	191.74 ± 28.76	217.83 ± 30.74	<0.001	199.57 ± 31.58	184.27 ± 30.8	<0.001
LDL-C (mg/dL)	96.54 ± 24.32	121.5 ± 26.88	<0.001	104.03 ± 27.5	94.58 ± 25.43	0.004
HDL-C (mg/dL)	45.89 ± 7.88	45.93 ± 7.58	0.97	45.9 ± 7.76	47.43 ± 6.84	0.203
TC/HDL-C	4.33 ± 1.16	4.9 ± 1.26	0.015	4.5 ± 1.22	3.98 ± 0.99	<0.001
LDL-C/HDL-C	2.19 ± 0.79	2.74 ± 0.93	0.001	2.36 ± 0.87	2.05 ± 0.69	0.002
hs-CRP (mg/L)	0.64 (0.34, 1.36)	2.31 (1.19, 5.52)	<0.001	0.83 (0.43–2.3)	0.55 (0.33–1.35)	0.009

Data is expressed as mean ± SD, except for hs-CRP level. *p* value was calculated at 95% confidence interval.

**Table 2:** Correlation of TSH levels of cases and controls with variables related to cardiovascular disease.

Variables	Subclinical hypothyroid (cases)		Euthyroid (controls)	
	(TSH >5 mIU/L)		(TSH = 0.25–5 mIU/L)	
	*R*	*p* value	*R*	*p* value
BMI (Kg/m^2^)	0.339	0.001	−0.033	0.748
Systolic BP (mmHg)	0.405	<0.001	−0.153	0.128
Diastolic BP (mmHg)	0.463	<0.001	−0.085	0.401
Total cholesterol (mg/dL)	0.572	<0.001	−0.118	0.243
LDL-C (mg/dL)	0.591	<0.001	−0.052	0.605
HDL-C (mg/dL)	−0.082	0.416	0.094	0.355
TC/HDL-C	0.378	<0.001	−0.12	0.235
LDL-C/HDL-C	0.451	<0.001	−0.076	0.452
hs-CRP (mg/L)	0.514	<0.001	−0.155	0.124

*p* value was calculated at 95% confidence interval.
